# The Extraction of Teeth

**Published:** 1898-08

**Authors:** George B. Clements

**Affiliations:** Macon, Miss.


ARTICLE VII.

### THE EXTRACTION OF TEETH.

DR. GEORGE B. CLEMENTS, MACON, MISS.

Read before the Mississippi State Dental Association, Jackson, April 7th, 1898.

Some two years ago I came to the conclusion that the extraction of teeth, as performed by the dental surgeon, was an unfinished operation if ever there was one from a scientific and surgical point of view. When the general surgeon is called upon to make an amputation, after the removal of the servered portion of bone he trims up and smooths off all jagged portions from the fractured end, and then folds his flaps over and stitches them down neatly and smoothly. I claim that after the extraction of teeth the gums should be turned back and the jawbone put in the same condition as is done by the general surgeon in case of an amputated limb; all ragged edges of the alveolus and the septa between the teeth should be trimmed down and a smooth surface left, over which the gums would heal rapidly. It is unscientific to leave the operation in an incomplete, unfinished state, causing the patient to suffer for weeks and weeks as the gums are drawn over those sharp, jagged points of broken alveolar process. When you extract a number of teeth you say to the patient: "After three or four months I will put in a permanent set of teeth, but you will have to wait for the gums to heal." And in the meantime, while the gums are "healing," scar tissue is formed, which is drawn tightly and rigidly over the projecting points of process, and often the patient will return, saying that you have broken off a root and left a projecting point which is cutting through the gum, and is very sore. You say, "Oh, that's all right; that's not a piece of root; let it alone, and in time it will be absorbed;" and you prescribe a mouth wash, and the patient goes home to suffer for weeks and months perhaps with a sore mouth, before the process is entirely absorbed. But I propose, in a few minutes, with a special forceps which I have devised, to trim the alveolus into just the shape I want it to have when ready for the permanent set of teeth. I propose to do in a few minutes what it takes nature, unaided, weeks, and sometimes months, to do.

When you are ready to extract the teeth put fifty drops of aromatic spirits of ammonia in a wine glass of water. This is a reliable, diffusible stimulant. Give it to the patient, and when you see a flush upon the face take out the teeth, then immediately pass the beak of this alveolar amputation forceps right in and clip out the middle septum, pass the flat side in between the periosteum and the bone and clip off all that is necessary to leave a smooth, rounded surface. After the shock of extraction the patient will not mind this minor operation if it is done immediately. In many cases there will be very little to remove; but whether much or little, you want it away from there; and if you do not remove it, you will have to wait for it to be absorbed. You will have an even surface over which to place your gum sections, and will not have to grind them out to fit over projecting places. In a very few days the gums will have healed nicely, and in from two or three weeks, at most, you can put in a permanent set of teeth, and you can put in block teeth as easily and as nicely as plain teeth. Patients will readily appreciate the advantage of this process over having to go for months without teeth, waiting for the gums to heal.

Another advantage in this method is that there will not be nearly so much subsequent absorption as when the stage of inflammation is continued weeks and months, for as long as inflammation continues the elements of the alveolar process are being absorbed and carried off. You will find that you will have but little subsequent absorption, and your permanent plate will be permanent. You will find this practice will be of benefit to yourself, to your patients, and I am sure it will be so to the profession. I thank you for your kind attention.—*Dental Headlight.*

